# School readiness and prematurity – a perfect storm?

**DOI:** 10.1038/s41390-025-04648-z

**Published:** 2025-12-01

**Authors:** Neil Marlow

**Affiliations:** Emeritus Professor of Neonatal Medicine, UCL Elizabeth Garrett Anderson Institute for Women’s Health, University College London, London, UK

Commentary on Morales M-F, Susperreguy M-I, Sims V. The long-term effect of very preterm birth on school readiness: Exploring preschool mediating pathways. *Pediatric Research 2025*

Morales and Colleagues report a study of school readiness in children from a large cohort of the general population concentrating on very preterm children (VP; <32 weeks of gestation).^1^ They chose outcomes at 8–12 years in areas which are frequently reported to be challenged in preterm cohort studies, and evaluated the mediating role of executive and receptive language at 3–6 years (pre-school). They observed lower maths skills in the VP group but no association with behavioural or emotional difficulties that were not explained by general cognitive scores. Preschool measures partially mediated the findings at 8–12 years. They suggest that attempts to enhance executive functioning and receptive vocabulary pre-school may mitigate the adverse effects of VP birth.

The size of the discrepancy is not as large as expected from other work in the literature but looking at the derivation of the index population gives clues as to why this may be the case. The classification as VP or term birth was derived from maternal self-declared gestation, without any confirmation of obstetric history. Thereby hangs a major issue, as the mean birthweight for this group is 2.86 kg and gestational age 28–29 weeks; this very high mean birthweight suggests a very significant recall bias and may explain the weak effects seen. Recall of gestational age is likely to be worse than that of birthweight. In one of our other VP studies, mean birthweight was 1.22 kg.^2^ This has the effect of diluting the effects seen in the current study but not the message.

It is very easy to identify potential areas for intervention, but delivering these interventions is much more challenging. Furthermore, there is much more to being ready for school than the broad brush of executive function and language. In many ways the broader physical, social and emotional skills are those that will determine whether a child is ready to go to and thrives in school, and it would be very helpful to identify areas where a child may need extra support so that a bespoke programme can be executed soon after commencing school, to mitigate areas of weakness or poor function. It is for this reason that the UK National Institute of Health and Care Excellence (NICE) recommends neonatal intensive care units extend follow up beyond 2 years (when impairments are simply counted up as a measure of the effectiveness of neonatal care), to the preschool period, where more predictive and diagnostic assessments can identify the detail of the issues faced by children and their families.^3^ The next step is critically important too – to then pass on these results to schools so that educators can identify children with aspects of the complex encephalopathy of prematurity and start to address them. The lack of success in this area is palpable in the UK, which is disappointing, and reports of the proportion of children having preschool assessment remain very low. As any parent will tell you the period of starting school and ensuring appropriate support is highly stressful. Given the body of work in this area school readiness assessment, which is not practised in the UK, is an important opportunity.

In addition to reduced cognitive scores, very preterm children may have a specific behavioural phenotype comprising inattentive-type ADHD, internalising behaviour and poor social communication skills, which is a constant over a range of studies and may be difficult to identify in a large busy classroom. Because of these behavioural and cognitive measures, it would be advantageous if parents identified their VP child as such to the school but sadly this rarely seems to happen.

Of course, it is also critical that teachers understand the potential differences between challenges in the several areas that differentiate very preterm and term births, respectively. The evidence from the UK^4^ and from the USA^5^ is that only a minority of teachers understand the different spectrum of learning needs of preterm children. The development of an on-line educational packages for teachers^6^ has been highly successful in raising this issue in the UK, reaching 32,000 downloads by parents and educators since it was released in 2018 (Samantha Johnson, personal communication) and is highly valued (https://osf.io/preprints/psyarxiv/48hmz_v1). Providing parents and teachers with key information at school entry may go a long way to minimising the impact of entering the educational system and ensure the system readiness to provide appropriate input.

Is there evidence that we can ameliorate these issues prior to school? There have been multiple attempts at neurodevelopmental enhancement, from the neonatal unit forward. Trials of family centred care have proven beneficial in some areas (cognition and behaviour) but not others, and have not extended out to childhood.^7^ This is important because the much larger trials of developmental intervention over the first 2–3 years showed promising early results that washed out after the intervention had ceased.^8^ Clearly, showing long-term efficacy of interventions is expensive and challenging to do, but it is critical to having the evidence for widespread implementation.

Might the interventional effort undertaken to date have the wrong target? Starting interventions in infancy would seem right and starting on the NICU even better. We are exhorted to intervene early. However, the specific areas of executive function and language that are affected in VP populations are actively differentiating in the third year and the pre-school period. The washout of effects over this period would imply that what is happening at that age may swamp the benefit of the early advantage. Indeed, there is interest and evidence that interventions targeting self-regulation and executive functions can be enhanced over this period and underpin academic and socioemotional competence.^9^ Perhaps it is time to look at the preschool period and determine how best to focus interventions to enhance development. The elusive goal of trying to prevent or reverse some of the developmental disadvantages seen in our very preterm graduates may then be within reach.
